# Breast Tomographic Ultrasound: The Spectrum from Current Dense Breast Cancer Screenings to Future Theranostic Treatments

**DOI:** 10.3390/tomography10040044

**Published:** 2024-04-15

**Authors:** Peter J. Littrup, Mohammad Mehrmohammadi, Nebojsa Duric

**Affiliations:** 1 Department of Imaging Sciences, University of Rochester, Rochester, NY 14642, USA; mohammad_mehr@urmc.rochester.edu (M.M.); nebojsa_duric@urmc.rochester.edu (N.D.); 2 Delphinus Medical Technologies, Inc., Novi, MI 48374, USA

**Keywords:** reflection, sound speed, attenuation, stiffness fusion, theranostic, time-reversed acoustics, hyperthermia

## Abstract

This review provides unique insights to the scientific scope and clinical visions of the inventors and pioneers of the SoftVue breast tomographic ultrasound (BTUS). Their >20-year collaboration produced extensive basic research and technology developments, culminating in SoftVue, which recently received the Food and Drug Administration’s approval as an adjunct to breast cancer screening in women with dense breasts. SoftVue’s multi-center trial confirmed the diagnostic goals of the tissue characterization and localization of quantitative acoustic tissue differences in 2D and 3D coronal image sequences. SoftVue mass characterizations are also reviewed within the standard cancer risk categories of the Breast Imaging Reporting and Data System. As a quantitative diagnostic modality, SoftVue can also function as a cost-effective platform for artificial intelligence-assisted breast cancer identification. Finally, SoftVue’s quantitative acoustic maps facilitate noninvasive temperature monitoring and a unique form of time-reversed, focused US in a single theranostic device that actually focuses acoustic energy better within the highly scattering breast tissues, allowing for localized hyperthermia, drug delivery, and/or ablation. Women also prefer the comfort of SoftVue over mammograms and will continue to seek out less-invasive breast care, from diagnosis to treatment.

## 1. Introduction

This review of breast tomographic ultrasounds (BTUSs) provides insights to the origins and clinical development of SoftVue (Delphinus Medical Technologies, Inc. Novi, MI, USA; DMT). The overarching questions we address are:

*#1. Why did SoftVue get Food and Drug Administration (FDA) approval for dense breast screening [1], especially in relation to its detection specificity; and*

*#2. Why is SoftVue NOT like any other current medical breast ultrasounds, such that its quantitative transmission parameters can also provide future breast cancer-targeted therapies?*

First, some insight into how long SoftVue has been in development, working toward a spectrum of clinical uses.

Historical Perspective of SoftVue: From the beginning, SoftVue was NOT intended to be just another form of automated breast ultrasound (ABUS). SoftVue was originally developed from meetings between several branches of the National Laboratories at the Barbara Ann Karmanos Cancer Institute in 1999, resulting in the first two patents utilizing SoftVue BTUS [2,3]. A driving premise was to take advantage of Moore’s Law, noting that a computer processing unit’s (CPU’s) speed and power were advancing exponentially. A primary technical goal was to acquire and analyze the huge amounts of 3D acoustic data coming from a circumferential field of insonified tissue, then to harness the greater processing speeds of the graphic processing units (GPUs) that arose from the burgeoning videogame industry. Increased processing speeds made BTUS scanning and image processing times a clinical reality that launched DMT in 2010. The current SoftVue unit contains eight GPUs and could also facilitate the rapidly advancing field of artificial intelligence (AI), which has demonstrated improved mammographic mass detection and tissue differentiation that are beyond that of breast radiologists [4,5]. The new quantitative transmission US parameters of sound, speed, and attenuation emphasized tissue characterization, and included its theranostic uses in a 2003 patent submission, awarded in 2013 [6]. Our initial focus has driven SoftVue diagnostics to the clinical reality of dense breast screening.

As a new clinical breast imaging modality, the purpose of this review is to first provide a primer for its current clinical use as radiologists embrace dense breast cancer screening. We believe it is also important to place this large volume diagnostic application in the perspective of its future therapeutic uses, thereby making SoftVue an overall potential solution for the large number of women with dense breasts requiring screening and the subsequent treatment of early, more curable breast cancers.

## 2. SoftVue—Breast Density, Risk, Screening and Specificity

**Figure 1:**
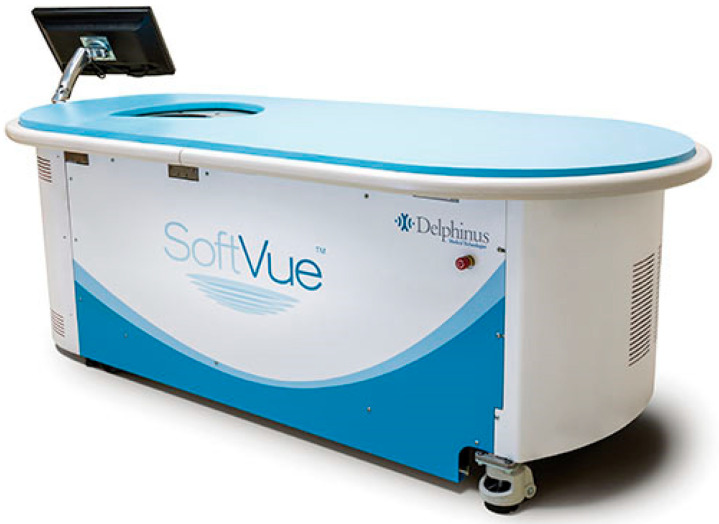
The SoftVue BTUS unit uses a prone water bath and ring array to scan in the coronal plane.

The SoftVue™ BTUS in Figure 1 received FDA pre-market approval (PMA) as an adjunct to mammography for dense breast cancer screening [1]. SoftVue is also the only form of ultrasound that has been FDA-approved as an adjunct to screening mammography in women with dense breasts at the time of the mammography. The initial SoftVue screening trial showed a 20% improvement in sensitivity and an 8% improvement in specificity when interpreted in combination with mammography [1]. The SoftVue PMA reader study found significantly more cancers with less false positives than full field digital mammography (FFDM) alone. Moreover, results from the patient experience survey conducted with participants in the screening trial found that women felt the gentle stabilization of the breast in a warm water bath was much more comfortable than mammography and that they would recommend SoftVue to other women. Breast density, cancer risk, screening specificity and other PMA approvals for breast cancer screening are briefly considered before highlighting the SoftVue imaging characteristics that improve clinical interpretation. 

### 2.1. The Density Risk 

Breast imagers and radiologists are facing a pivotal challenge of providing cost-effective, practical screening solutions for women with dense breasts who have well-established greater risks of both developing breast cancer, and having it missed by mammography. The progressively increasing breast cancer risk of a breast imaging study outcome, 1–5, has been detailed in the most recent 2013 update to the Breast Imaging Reporting and Data System (BI-RADS) [7]. A recent meta-analysis, using the most up-to-date BI-RADS density designations (i.e., densities a–d) and FFDM, rather than a film screen, confirmed the ~2-fold higher risk of developing breast cancer in women with overall dense versus non-dense breast tissues (i.e., BI-RADS c–d versus BI-RADS a–b) [8]. Moreover, when women with extremely dense breasts (BI-RADS d) are compared to women with fatty breasts, the risk may be >6-fold, and >50% of cancers may be missed and/or hidden by the dense parenchyma [9]. However, screening also needs to be efficient and cost effective.

### 2.2. Specificity and DBT

Practical workflow and societal benefits have been seen with other screening advancements, such as digital breast tomosynthesis (DBT). Also known as 3D mammography, DBT allows the partial visual removal of overlying dense breast tissues for a better evaluation of potential underlying masses, architectural distortion and/or suspicious calcifications. The major benefit that DBT offered over FFDM was the reduction of false positives and the associated unnecessary biopsies. Indeed, the FDA PMA for DBT (GE, SenoClaire, GE Healthcare, Chicago, IL, USA) actually showed slightly lower sensitivity (i.e., 4.5% lower, or 0.831 versus 0.7864 for the sensitivity of FFDM versus DBT, respectively), which was acceptable for this non-inferiority study [10]. More importantly, DBT showed a 6.5% higher specificity (i.e., 0.67 versus 0.736) and a 6.6% lower recall rate (i.e., 0.406 versus 0.340) than FFDM. Similarly, SoftVue’s PMA performance over FFDM showed a 20% improvement in sensitivity and an 8% improvement in specificity [1]. 

The improved clinical specificity drove DBT and “3D mammography” to become the standard-of-care for a screening mammography in most breast centers throughout the United States. Specificity has been a primary focus for screening outcomes for over two decades, when it was shown to be the crucial parameter that drove screening outcomes in a landmark benefit–cost analyses for the American Cancer Society’s National Prostate Cancer Detection Project [11]. It is not surprising that DBT has also shown encouraging cost-efficacy evaluations [12] due to its reductions in both mammographic callbacks and unnecessary biopsies. Therefore, most insurance companies now cover DBT-related breast cancer screenings and diagnostic evaluations. 

### 2.3. Specificity and Breast Ultrasound 

The predicate for SoftVue’s FDA PMA was GE’s ABUS device, Invenia [13], which is similar to the extended field-of-view ABUS and the hand-held ultrasounds (HHUSs) produced by other companies. Current breast ultrasound (HHUS and ABUS) screening studies have provided an ~20% increase in sensitivity, but the clinical adoption of US breast screening has been slow due to its overall decrease in specificity and the clinical burden of managing its associated follow-ups. For example, GE’s Invenia PMA showed that sensitivity increased by 23.7% when Invenia ABUS was combined with FFDM (62.4%) compared to that of FFDM alone (38.5%) [13]. ABUS provided an improved clinical workflow with operator independent scanning and a similar sensitivity, yet these benefits were mitigated by the clinical burdens of false positives and the associated biopsies, just like with HHUS. GE Invenia received PMA approval for dense breast screening as an adjunct to mammography, but their indication required a radiologist to first provide a negative/benign BI-RADS score of 1 or 2 on a mammogram before an ABUS exam could technically be performed. SoftVue’s indication offers the advantage of being performed with mammography and not needing a radiologist to first interpret the mammogram (i.e., assuming no off-label uses). SoftVue’s specificity makes it unique for the US screening of dense breasts.

### 2.4. What about Other Imaging Modalities for Dense Breast Screening?

Over 27 million women, aged 40–74, in the US alone have dense breasts (e.g., >40%) and there remains a burgeoning need for cost-effective, practical imaging solutions [14]. Several other imaging modalities provide an increase in the incremental cancer detection rate (ICDR; i.e., additional cancers per thousand women screened), or an improved sensitivity over FFDM alone, but also have significant societal and cost-related concerns. Some of these favorable ICDRs are difficult to compare with patient populations having different baseline incidence rates (i.e., family history, BRCA1/2, etc.) [14]. However, the purpose of this review is not to compare SoftVue to other modalities that do not have an FDA approval for dense breast screening. We highlight the practical use of SoftVue as a new non-ionizing, non-invasive imaging technology that extends beyond its screening PMA. SoftVue’s additional 510 (k) FDA clearances also address several diagnostic uses for SoftVue in patients with all breast densities, and are covered under current ultrasound billing codes. 

Despite the recent and continued interest in contrast enhanced mammography (CEM), molecular breast imaging (MBI), and breast magnetic resonance (MR), none have completed an FDA PMA trial as an adjunct to screening mammography in women with dense breasts. Extending the diagnostic performance of CEM, MBI, or breast MR from limited high-risk groups to the millions of women with dense breasts remains a serious question requiring additional radiation (e.g., CEM and MBI) and intravenous contrast injection. Moreover, MR may be the current “gold standard” for breast imaging, but claustrophobia and other concerns caused ~40% of women to refuse a free breast MR as part of a research study [15]. Lower cost and more accessible screening is still needed, well beyond what MR could provide. We will now clarify the technical and clinical imaging aspects of SoftVue BTUS to foster the confident usage of this new breast imaging tool.

## 3. Understanding SoftVue Image Sequences: Tissue Acoustics and Clinical Imaging

### 3.1. HHUS Reflection vs. SoftVue Reflection 

**Figure 2:**
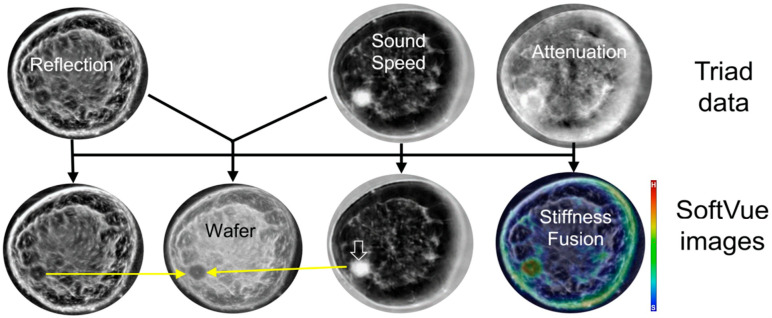
SoftVue scans each breast in the coronal plane, acquiring the data triad of reflection, sound speed, and attenuation (**top** row). Four SoftVue image stacks (**bottom** row) then consist of Reflection, Wafer (WAveForm Enhanced Reflection), Sound Speed, and Stiffness Fusion image stacks [23]. Arrows show the composite imaging of a sound speed-corrected reflection for Wafer and the combination of Sound Speed and attenuation data overlaid upon Reflection for Stiffness Fusion. The irregular 1.2 cm cancer at the eight o’clock position is bright on Sound Speed (open arrow), which then combines with the Reflection image to remain dark on Wafer (yellow arrows), while the fat on Wafer becomes gray and makes the cancer better seen than on Reflection alone for screening.

The power of SoftVue lies in its multiple image sequences, seen in Figure 2, which have been extensively covered in peer-reviewed publications and presentations over the past two decades [16,17,18,19,20,21,22,23,24]. Briefly, the SoftVue sequences allow for the initial heightened detection of masses by using Wafer and Sound Speed sequences, and then, if a suspected mass is found, its “persistence” on standard Reflection and its “hardness” evaluation by Stiffness Fusion help characterize the mass as noted below. The technical specifics of the SoftVue unit has been previously described [23]. Other groups have explored ultrasound tomography for over 40 years [25,26,27,28,29]. One group reached a clinically approved device [30,31,32,33,34], but it does not provide a stiffness evaluation of masses, or the whole breast, and has not received FDA PMA approval for dense breast screening. We only highlight our results with SoftVue due to its extensive clinical documentation [16,17,18,19,20,21,22,23,24]. Relative to standard breast ultrasounds, SoftVue relies on the quantitative transmission parameter of sound speed (SS) and attenuation (ATT), but the differences between the reflection imaging performed by HHUS/ABUS and SoftVue are notable.

As radiologists, we have grown accustomed to interpreting HHUS signal aberrations and processing artifacts. Standard US beam forming, signal generation and image processing using a hand-held array produces a signal whose reflections are then registered by the remaining transducer elements. Using a ring array for SoftVue Reflection, a single transducer emits an acoustic signal that is then registered by all the surrounding elements, generating both full-field reflected acoustic signals, as well as the transmitted data across the ring. 

**Figure 3:**
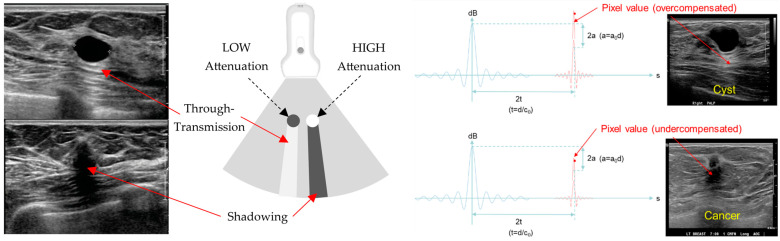
(**left**) and (**right**): Standard US showing a low-attenuation cyst (**top left**) with posterior through-transmission (**top**) and a high attenuation cancer causing posterior shadowing (**bottom**). When identical emitted acoustic waves (blue lines) and the resultant received signal (red lines) are assumed to occur, a uniform acoustic medium overcompensation of the anticipated signal posterior to a cyst occurs due to the greater signal strength from low attenuation (**top right**). Shadowing, or the under-compensated signal strength behind an attenuating breast cancer thus occurs from the less-than-anticipated signal strength posterior to the cancer (**bottom right**).

SoftVue’s Reflection image stack uses an extensive circumferential process that eliminates the artifacts we have become accustomed to, such as the “shadowing” and “through-transmission” noted in Figure 3, and allows for an accurate view of coronal cross-sectional breast anatomy. SoftVue Reflection will thus not look like the compensated, artifact-ridden reflection images from high frequency B-mode HHUS. The acoustic waves from the ring array insonate all margins of a mass in a 360° SoftVue Reflection algorithm. Moreover, the ring array allows for the anatomic co-registration of the coronal SoftVue image sequences for the localization and characterization seen in Figure 2. The coronal imaging axis has also shown a superior depiction of the architectural irregularities caused by breast cancers [35,36].

Compared to the high-frequency HHUS pulses of up to 20 MHz, a 3 MHz transducer pulse was chosen for SoftVue to allow adequate tissue penetration to fully insonate breast tissues across the 22 cm SoftVue ring array, yet provide the submillimeter resolution needed for detailed mass margin assessments and the BI-RADS characterization of breast masses. Attenuation decreases with decreasing frequency. At around 3 MHZ, ultrasound signals can penetrate the whole breast, a requirement for SoftVue imaging. Decreasing frequency also means decreasing resolution. So, 3 MHz is the sweet spot for the high resolution (submm) imaging of the entire breast. SoftVue Reflection achieves submillimeter resolution by removing speckle, clutter, and noise, similar to the compound imaging for the B-mode HHUS. Image resolution is also dependent on the grid size and resultant computational time or burden on the eight GPUs. 

### 3.2. SoftVue Sound Speed

The speed of sound is a direct correlate of breast density [17,18,19,20], and benign masses are often hidden by parenchyma, while cancers may have even higher densities. Similar to the tomographic nature of DBT allowing for better detection and differentiation of adjacent dense tissues, SoftVue helps separate dense parenchyma from similarly dense underlying masses in a 3D format. But, the separation of fat from all other breast tissues remains clearly defined, whether that be via mammographic or sound speed breast density. The sequential display of coronal breast density in SoftVue’s Sound Speed image stack shares more similarities to reviewing DBT, X-ray computed tomography (CT), or MR. The dense fibroglandular tissues encountered in ~40% of women may thereby partially or completely obscure underlying masses of similar densities. 

The prone coronal 3D SoftVue Sound Speed image stack minimizes tissue overlap better than a single 2D mammogram, or even a stack of compressed DBT images. The quantitative parameters of Sound Speed and Attenuation also help define relative tissue differences and thus the conspicuity of adjacent tissues. Table 1 shows the greatest relative percentage difference between the highest sound speed material in a phantom study, whereby the sound speed of a gelatin cyst (1585 m/s) is only 7.4% (118/1585) greater than that of fat (1467 m/s), the lowest sound speed material tested [37]. Moreover, there is only a 0.7% (11/1563) higher sound speed of the cancer than of the parenchymal tissue (1552 m/s) which can obscure it. Conversely, quantitative attenuation differences at even 2.5 MHz can demonstrate much greater relative differences between most tissues (i.e., a 25.8% difference between cancer and parenchyma, or 1.20 and 0.89 dB/cm, respectively). The human eye is sensitive to large relative image differences, which is why mass detection and characterization within dense breast tissue is more effective when using the combination of Reflection, Sound Speed, and Stiffness Fusion images that incorporate Attenuation values (Figure 2) [23]. 

**Figure 4:**
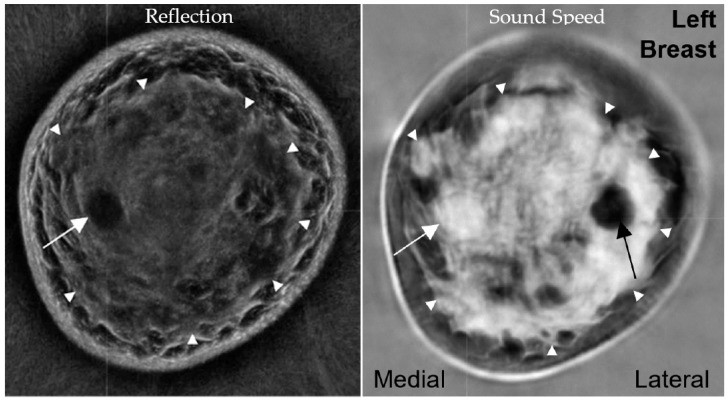
A 52-year-old woman with extremely dense breast parenchyma and a 1.6 cm fibroadenoma (white arrow) in the **left**, lower, inner quadrant at the FGI (arrowheads). The fibroadenoma is more conspicuous in the Reflection image (**left**), while the Sound Speed image (**right**) shows that the mass abuts fat on only a narrow peripheral margin. A fat lobule surrounded by parenchyma creates a pseudomass on Sound Speed (black arrow, **right**), whereas Reflection shows the underlying architecture, like the sub-dermal fat lobules beyond the FGI. (Data source: Original case from DMT’s Prospective Case Collection trial; Western IRB #DMT-2015-001).

In our experience, the FGI is well seen by the Sound Speed and Reflection sequences noted in Figure 4 and was the origin of >95% of breast cancer locations [22]. Previously noted MR descriptions of this phenomenon also emphasized the likely underlying pathophysiology of greater hormonal and/or growth factor influences at the FGI [38,39]. While ~63% of fibroadenomas in our series were also commonly located at the FGI, 64% of cysts and 25% (37/105) of fibroadenomas were completely surrounded by dense tissue [22]. However, no cancers were noted to be completely surrounded by dense tissue. The FGI can thus be used as an additional visual criterion to guide the cancer detection performed by radiologists and serve as an input for future machine learning algorithms. 

### 3.3. SoftVue WAveForm Enhanced Reflection (Wafer)

As previously noted, Wafer was conceived to boost the background fat signal in the Reflection images to better differentiate dark masses from the normal dark fat lobules seen on Reflection. Wafer embodies two functions. The first is to remove fat signals by using information from the sound speed image to mitigate the hypoechoic nature of fat at these frequencies. It is essentially a form of fat correction which makes masses more conspicuous. The second function is to enhance masses further by taking the gradient of the sound speed image, which under a constant density assumption represents changes in acoustic impedance which serves to enhance mass edges. The combination of these actions generates more contrast for mass detection. In other words, by dividing Reflection pixel values by the concurrently registered Sound Speed values, the lower values of fat and the higher values of true solid masses effectively created brighter Wafer pixel values for fat and darker relative appearances of solid masses, respectively. Wafer increases or brightens the fat signal and does not subtract it, effectively “suppressing” breast fat from being mistaken as a suspicious dark mass on Reflection. In other words, Wafer is more appropriately called a fat suppression technique.

**Figure 5:**
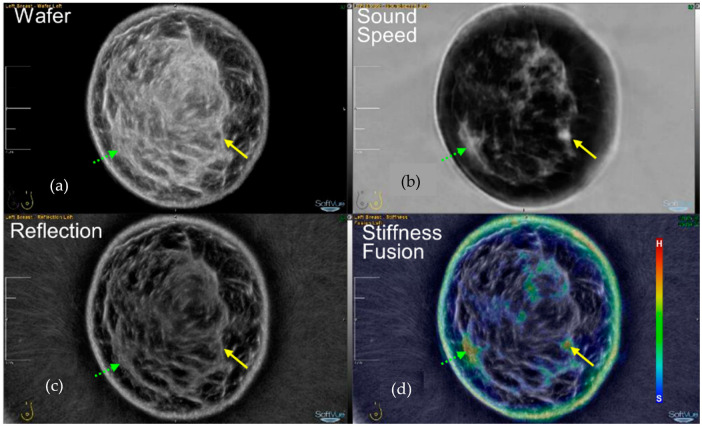
The 4 SoftVue image stacks (**a**–**d**) show the improved conspicuity of the small (<1 cm) cancer (yellow arrows) at the 4:00 position on Wafer (**a**), Sound Speed (**b**), and Stiffness Fusion (**d**), compared to its nearly undetectable Reflection (**c**) appearance. This is facilitated by the cancer’s high-density bright appearance on Sound Speed (**b**) in a predominantly fatty breast, making the cancer appear darker on Wafer. However, the subtle “persistence” of some darkness on the Reflection image (**c**) helps differentiate it as a true mass, unlike the dense parenchyma (green dashed arrows), which remains bright on Reflection. A region of residual bright parenchyma (green dashed arrows) appears to be amorphous and helps dismiss it as false positive stiffness (red) rather than an actual mass on Stiffness Fusion. BI-RADS 4c (irregular/indistinct + red). (Data source: Original case from DMT’s Prospective Case Collection trial; Western IRB #DMT-2015-001).

Figure 5 demonstrates numerous aspects of SoftVue screening detection and the subsequent characterization of a sub-centimeter cancer from the DMT multicenter screening study [1]. First, Wafer and Sound Speed are paired as two sequences that are scrolled through in tandem for the optimal initial detection of the small, bright (high density) cancer on Sound Speed, and the small dark area along the FGI on Wafer (yellow solid arrows). Despite its almost imperceptible appearance on Reflection, once the cancer is better localized by Wafer, it can be seen to “persist” as a relatively dark region on Reflection. This helps define it as a true mass for further screening follow-up that is much different from the dense fibroglandular tissues which remain bright on both Wafer and Reflection (green dashed arrows).

Note that the Wafer image in Figure 5 likely depicts the original extent of fibroglandular tissue before it underwent fatty replacement with aging, whereby the Sound Speed image shows mostly fat at this level. Conversely, the Reflection image still retains plenty of echogenic surfaces throughout the breast that are not related to the high density parenchyma. In other words, Reflection may still retain those echogenic surfaces where the parenchyma used to be, then regressed and became volumetrically “filled in” by fat, which now brightens the central fatty regions on Wafer even more. As a non-ionizing breast imaging device, such as a historical display of breast tissue evolution and parenchymal regression over time, may be verified in future studies with younger women, such as in chemoprevention and dietary intervention studies. SoftVue breast density, risk, and treatment monitoring applications continue to evolve [17,18,19,20,21,24].

### 3.4. SoftVue Stiffness Fusion

The Stiffness Fusion image stack overlays stiffness maps (made by combining the quantitative transmission parameters of sound speed and attenuation), onto the Reflection image stack. SoftVue is also unique in providing the first tissue compressibility (or elastography-like) evaluation of the entire breast, which differentiates SoftVue from any other breast US technology. As we have noted [23], whole breast stiffness is a surrogate of the bulk modulus and defines a material’s resistance to compression from a longitudinal wave, which is used for all of SoftVue imaging. Pixel values in Stiffness Fusion are displayed on a color scale representing the full stiffness values found within a given breast and optimized for the relative stiffness differences in that breast. By varying the shape and strength of an acoustic wave, a larger dynamic range and greater tissue differentiation by the bulk modulus than the current strain or shear wave elastography may be possible [23].

Even in extremely dense breasts, only a small portion of the total breast volume (11.2%) may be considered hard by Stiffness Fusion, and ~80% of that comes from dense fibroglandular tissues (i.e., 9.0% of the total volume) [23]. Breast density and stiffness has been noted as a risk for cancer and the addition of SoftVue’s Stiffness Fusion image sequence may provide further whole-breast-relative risk characterization [40]. The most important utility of Stiffness Fusion is for characterizing breast masses, similar to elastography [23]. As cysts are more common than cancer, having the confidence to characterize a well-circumscribed mass as a simple cyst is crucial for screening specificity.

The general lack of attenuation from cysts produces very low “soft” values, making cysts appear with a minimal internal Stiffness Fusion signal (e.g., black/blue/green). Solid masses show a greater relative percentage of “hard” (e.g., red) components for masses ≤1.5 cm [23]. Cancers therefore have the greatest relative stiffness and fibroadenomas are intermediate. For the small solid masses frequently encountered during screening, soft components will make up to 50% of the composition of fibroadenomas, whereas cancers will have almost NO soft components. The tumor homogeneity of the stiffness images was noted to be significantly lower for cancers [23], which may be another feature for AI. An additional step in Stiffness Fusion characterization is determining if the color or stiffness is focal. With a true mass, the color will remain focal and stay within the mass margins. Hard fibroglandular tissue will not stay focal to the area of concern but pass over and around the area when scrolling through the image stack. In other words, smaller cancers are often the most focally stiff (red) region within the generally soft normal breast volume. This is best seen using the zoom feature to characterize the mass on all four sequences (see also Figure 8).

## 4. SoftVue UST and US BI-RADS—Review of Common Breast Masses

SoftVue generates multiple parameters to assess mass characterization. SoftVue’s Reflection mode is akin to B-mode and can use standard US BIRADS when it comes to echogenicity and shape [7]. On the other hand, the SoftVue sequences of Sound Speed and Stiffness Fusion have no counterparts in B-mode and needed new criteria. Thus, a comparison was needed to highlight both the familiar criteria as well as the new criteria. The sequential SoftVue characterization of cysts, fibroadenomas, and cancers also uses the US BI-RADS parameters of mass shape and margin (Table 2).

Table 3 summarizes the increasing level of suspicion noted for BI-RADSs 1–5 [7].

### 4.1. Cysts on SoftVue

**Figure 6:**
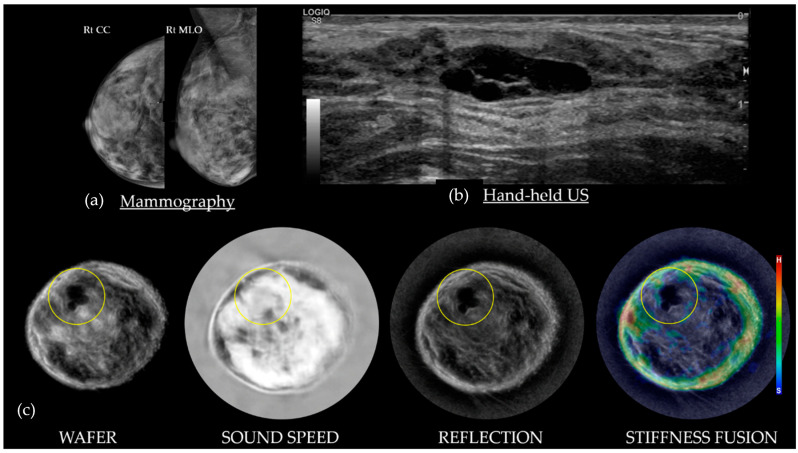
A 54-year-old with extremely dense breasts on mammography (**a**) and a palpable cluster of simple cysts on subsequent HHUS (**b**) in the 11:00 upper outer quadrant of the right breast. The bottom row of SoftVue BTUS image stacks (**c**) show black appearances of the lobulated 2 cm cyst on both Wafer and Reflection, intermediate (gray) appearance on Sound Speed, and no color on Stiffness Fusion. BI-RADS 2. (Data source: Original case from DMT’s Prospective Case Collection trial; Western IRB #DMT-2015-001).

Figure 6 shows simple cysts as dark on Wafer, usually gray on Sound Speed, black/persists on Reflection, and black/blue on Stiffness Fusion. As cysts are composed primarily of water and have minimal attenuation, these masses appear as blue or void of any color on Stiffness Fusion. Cysts are generally surrounded by fibroglandular tissue [22].

### 4.2. Fibroadenomas on SoftVue

**Figure 7:**
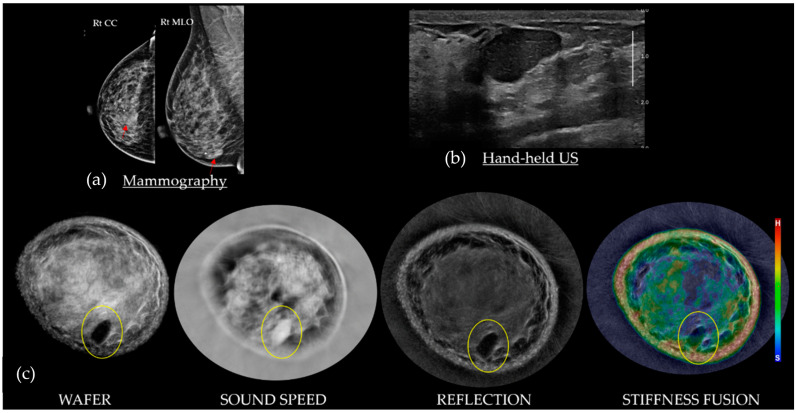
A 48-year-old with heterogeneously dense breasts and an ovoid 2.5 cm mass on the right mammograms (**a**; red arrows). An ovoid relatively homogeneous solid mass corresponding to the mammographic and palpable abnormality, seen on HHUS in the 6–7 o’clock position (**b**). The bottom row of SoftVue BTUS image stacks (**c**) shows that the fibroadenoma (yellow circles) has subtle echoes on Reflection, which become black on Wafer due to the high Sound Speed correction, and are an intermediate color on Stiffness Fusion. BI-RADS 2. (Data source: Original case from DMT’s Prospective Case Collection trial; Western IRB #DMT-2015-001).

Figure 7 shows that most FAs are dark on Wafer, white on Sound Speed, and persistent on Reflection. The stiffness of a fibroadenoma can be quite variable, relative to the extent of underlying fibrosis, ranging from a hard scirrhous mass (i.e., red) to a soft parenchymal mass (i.e., blue-green). Fibroadenomas generally remain circumscribed on Stiffness Fusion, with their color(s) remaining within the margins of the mass while scrolling. Next to cancers, fibroadenomas are the most frequent mass at the fat–glandular interface (FGI), but they can also be surrounded by parenchyma [22].

### 4.3. Cancers on SoftVue

**Figure 8:**
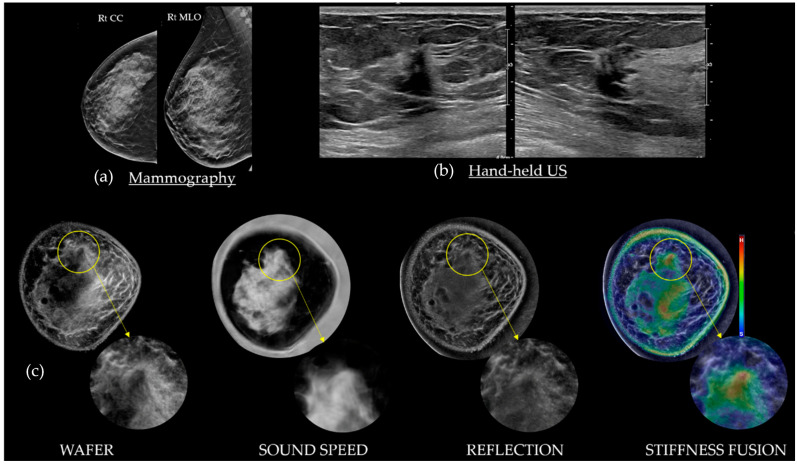
A 63-year-old with heterogeneously dense breasts and a normal mammogram (**a**). An irregular, taller-than-wide, shadowing, 0.8 cm cancer seen by HHUS corresponded with the palpable abnormality in the 12 o’clock position of the right breast (**b**). The bottom rows of SoftVue BTUS image stacks (**c**) show standard and zoomed appearances of the cancer (yellow circles), crucial for margin assessment. Similar to mammography, Sound Speed images can partially obscure the mass within the high density adjacent parenchyma. However, this high density cancer is better seen as a dark spiculated region on Wafer by darkening the subtle gray area that “persists” on Reflection, unlike that of the dense parenchyma. Finally, Stiffness Fusion shows focal stiffness (i.e., red) limited to that suspicious mass. Of note, the other central, lower, stiff (red) region appears as a brighter parenchyma on both Reflection and Wafer. BI-RADS 5 (red + spiculations). (Data source: Original case from DMT’s Prospective Case Collection trial; Western IRB #DMT-2015-001).

Figure 5 and Figure 8 show that most invasive breast cancers are irregular in shape with indistinct or spiculated margins, dark on Wafer, bright on Sound Speed, and stiff (red) on Stiffness Fusion. Cancers can be round or oval, similar to standard US (e.g., medullary, papillary, or mucinous types) but more work is needed for further sub-classifications of cancer. Associated architectural distortion or peritumoral edema may be observed, especially in the native coronal plane of SoftVue, along with spiculation as noted by others [35,36]. The fibrotic cicatrization surrounding many cancers (e.g., those that are ER/PR positive) may display the surrounding tissue’s interactions with the terminal ductal lobular units, where cancers originate. Approximately 95% of cancers arise at the FGI, likely due to their biological stimulation from the adjacent subcutaneous fat, now considered to be a potent endocrine source [22]. Any mass with spiculations or architectural distortion is consistent with cancer as long as it does not correlate with a known area of surgical scarring. Invasive breast cancers also vary in appearance based on their size. Small cancers tend to present with more central stiffness which routinely appears as a completely red or orange mass on Stiffness Fusion. Larger cancers (i.e., >1.5 cm) are likely to be softer and appear as green or mixed on Stiffness Fusion [23].

### 4.4. SoftVue and BI-RADS

The process of detecting and characterizing a mass using SoftVue can be placed into a lesion characterization matrix, incorporating applicable US BI-RADS criteria [7] and the previously noted BTUS-specific parameters (Table 2). Table 3 summarizes the increasing level of suspicion noted for BI-RADSs 1–5, noting the accelerating likelihood of cancer from BI-RADSs 4A–5. This increasing cancer risk has been noted in prior studies.

**Figure 9:**
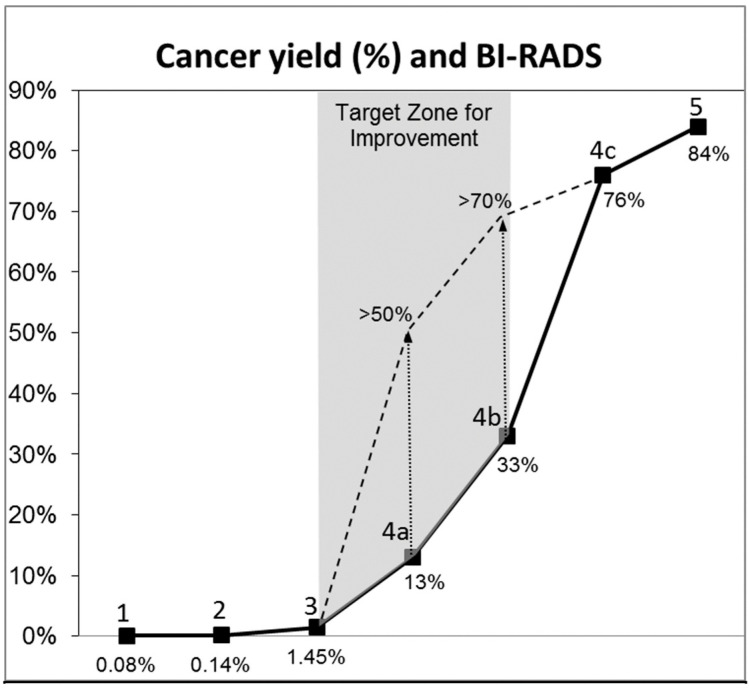
The solid line notes the increasing risk of cancer noted with increasing BIRADs from 1 (negative) to 5 highly suspicious), similar to Table 3. The target zone for biopsy yield improvement (gray) = the steeper inflection between BI-RADSs 3 and 4b.

An overall goal for SoftVue becoming part of routine breast imaging would be to improve the positive predictive value (PPV) of cancer on biopsy outcomes as postulated in Figure 9. In other words, the “art” of breast imaging and diagnosis may reside at the “target zone for improvement”, mainly between BI-RADSs 3–4b, which currently represents a composite of cancer detection rates [41] and the PPVs of BI-RADSs 4a–c for mammography and US [42,43,44]. We believe Table 3 functions within that target zone to reduce call-backs and unnecessary biopsies, but additional clinical data will further refine these diagnostic criteria.

Figure 9 reminds us of the need to continue diagnostic efforts to provide optimal patient care to millions of women as SoftVue screening continues. Further improvements in patient outcomes from these large efforts suggest the need for the continued expansion of machine learning and advancements in computer-aided detection (CADe) and diagnosis (CADx), which may better utilize the multiple quantitative parameters of BTUS in the near future.

## 5. The Future of Dynamic Focusing for Therapy

**Figure 10:**
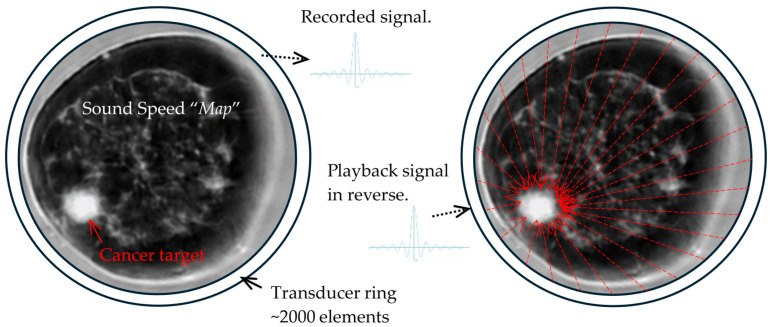
Dynamic focusing using time-reversed acoustics [46]: In the recording step (**left**), the SS maps generated by SoftVue are used to time the acoustic waves from each transducer element (~2000) to the cancer in the 8:00 position, as seen also in Figure 2. In the focusing step (**right**), the time-reversed signals are emitted from each element (red arrows—only 27 shown) such that they will all coalesce at the selected focal points throughout the extent of the cancer and up to 1cm beyond its margins. Accurate temperature monitoring by SoftVue tissue maps is enabled between each (or several) focusing shots, using the priori knowledge of the tissue composition (fat vs. water-based soft tissue).

The two acoustic capabilities of SoftVue that make its therapeutic uses for breast cancer very intriguing are its noninvasive temperature monitoring and its potential for an aberration-corrected, improved focusing of acoustic energy in highly heterogenous breast tissues (Figure 10) [45,46]. The accurate tissue acoustic maps (using sound speed and acoustic attenuation) acquired by BTUS provide a unique opportunity to fine-tune the time-reversal technique and generate a desired acoustic pattern for low-intensity focused ultrasound (LIFU) therapies such as mild hyperthermia [47,48] and potentially high-intensity focused ultrasound (HIFU) ablation [49].

Combined therapy and diagnostic functions, or theranostic potentials, have been generally limited to radiotracer/therapy molecules and nanoparticles [50,51], including ^177^Lu-trastuzumab (Herceptin), ^177^Lu-DOTATATE, and ^177^Lu-FAPI-46, specifically for breast cancer [52]. Ultrasounds have been used such as the high-intensity focused ultrasound (HIFU) for breast cancer ablation, yet the current HIFU is limited primarily to thermally sensitive MR-guidance and some attempts at standard US guidance in China [53]. Breast tissues are highly scattering and present challenges for the delivery of HIFU acoustic energy, with adherent risks of unintended skin and/or soft tissue burn complications.

SoftVue’s sound speed and attenuation maps can thus mitigate these risks by using a unique form of focusing known as “time-reversed” (TR) acoustics (Figure 10) that actually improves focusing in highly scattering breast tissues [45,46]. Figure 10 depicts the ring array of SoftVue but with far fewer emitted acoustic signals than in a current SoftVue ring array (i.e., 2048) [22,23]. Moreover, we now have the ability to do continuous temperature monitoring using SoftVue’s sound speed, using the average temperature change in tissues of ~2 m/s/°C, again using interleaved SS imaging sequences [47,48], similar to the MR monitoring of the HIFU [53]. Fat shows a uniquely different heating pattern than the breast parenchyma [54,55], with decreased SS up to 50 °C, but the SoftVue localization of fat is already well-defined by SS. Finally, attenuation has been noted to markedly increase following ablation and the associated protein denaturation [54] which can also be monitored by the Stiffness Fusion sequence as noted.

**Figure 11:**
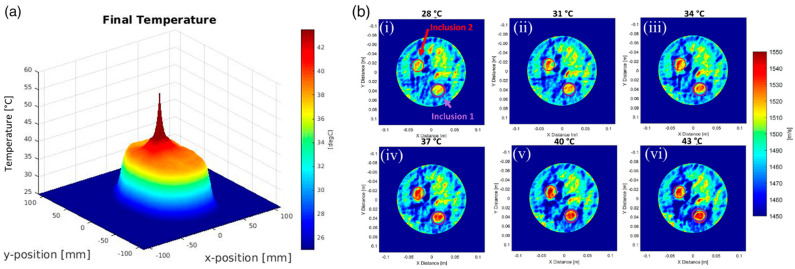
[47]: A series of 2D, in silico, mild hyperthermia therapy (MHTh) induction results for multi-focus showing: (**a**) a final temperature map demonstrating TR-focusing outcomes; and (**b**) SS images at all temperatures, (**i**) 28 °C, (**ii**) 31 °C, (**iii**) 34 °C, (**iv**) 37 °C, (**v**) 40 °C, and (**vi**) 43 °C, where both inclusions show a progressive increase in temperature in 3 °C increments from yellow to red.

Figure 11 demonstrates our ongoing work with both temperature sensing and dynamic focusing using modeling and prototypes [46,47]. Further work is needed to transition SoftVue, from an FDA-cleared imaging device for dense breast screening to a theranostic device, capable of the guided delivery of accurately focused acoustic energy to a known tumor/target volume. Acoustic energy is thus converted into volumetrically delivered mild hyperthermia, tailored to cover the SoftVue visualized tumor and/or target volume (e.g., breast biopsy markers, etc.). This first iteration of breast cancer hyperthermia has been shown to augment chemotherapy, radiation therapy and immunotherapy. The targeted delivery, or the controlled-release, of microbubble or nanodroplet chemo/immunotherapy [56,57], and both thermal- and non-thermal-targeted ablations are envisioned for this exciting new field of theranostic possibilities. Finally, the focusing power exists for TR acoustics to increase temperatures to ablative levels (e.g., >60 °C).

Our feasibility studies indicated that inducing hyperthermia with a ring-array ultrasound transducer does not add significant safety concerns and, thus, we envision a relatively easy regulatory path to adopt this technology in clinical applications. In a preliminary feasibility study, a 256-element ring-array was used to generate and sustain mild hyperthermia at a central focal point in a tissue-mimicking phantom made out of polyvinyl chloride (PVC) [47,48]. In order to generate heat in an average of 152 s, the maximum required pressure was measured as 800 kPa (peak negative pressure). This corresponds to a mechanical index (MI) of 0.65, which is significantly below the risk of adverse bioeffects such as cavitation (MI < 1.9 is the FDA recommendation for diagnostic devices) [58]. For reference, in standard mild hyperthermia treatment, where the target temperature typically does not exceed 43 °C [59,60,61,62], peak pressure typically ranges from 1–3 MPa (max intensity 10–100 W/cm^2^) depending on the desired rate of heating [62,63,64,65,66,67]. The acoustic pressure in a ring-array transducer was lower than others reported in the literature for generating mild hyperthermia using a focused ultrasound (FUS), which validates the efficacy and safety of the device. Given that the transmit acoustic power for each transducer element is kept in a low range and is safe for diagnostic imaging, it is anticipated that a ring-array can generate heat at a significantly faster rate with minimal risk by increasing the duty cycle or the voltage. In addition, the constant monitoring of tissue temperature during hyperthermia and using it as live feedback to adjust the system will further insure the safe operation of the system in its theranostic mode.

## 6. Conclusions

SoftVue’s FDA PMA approval [1] was focused on screening, and several additional 510(k)’s cover diagnostic imaging. The improved specificity of SoftVue while finding an anticipated 20% additional cancers holds promise for its cost-effective adoption as an adjunct to mammography for women with dense breasts. Additional studies are needed to convert this 20% increase in cancer sensitivity to a CDR, but it appears to be comparable to standard USs and ABUSs providing approximately additional 4 cancers per 1000 patients. The important increase in specificity translates into less patients undergoing unnecessary callback and/or low yield breast biopsies. The four different SoftVue image stacks using reflection and transmission imaging provide both improved mass detection for sensitivity, as well as the subsequent tumor characterization for improved specificity. The ability to incorporate new SoftVue parameters into the BTUS BI-RADSs 1–5 risk gradient allows for the practical mass classification for the appropriate current clinical workup of any suspected mass.

The future of SoftVue and therapeutic treatments is also encouraging. The cross-over from diagnosis to therapy has fortunately expanded into the image-guided treatment of breast cancer, as demonstrated by the Society of Interventional Oncology including the first master’s course in breast cryoablation as the leading ablation option in 2024. We participated in the pioneering development of cryoablation as a predominantly CT and/or US-guided tumor ablation following the inception of SoftVue, when we also performed the first percutaneous cryoablation of the liver in Guangzhou, China in 1999. We then performed the first breast cryoablation for multi-focal breast cancer in 2003 and published the first series of breast cancer patients who did not undergo subsequent surgical resection for breast cancer, with a mean tumor diameter of ~1.7 cm, but who had no apparent clinical or imaging recurrences for 18 months [68]. This clinical success was mostly dependent upon understanding the lethal isotherms that needed to be generated and sculpted by multiple cryoprobes to produce cytotoxic margins (e.g., <−30 °C) that extended beyond all apparent tumor margins [69]. This is the same process that will need to be transferred to future BTUS theranostic uses in order to deposit TR-focused acoustic energy with a similar volumetric coverage of SoftVue-defined tumor margins, whether that be for mild hyperthermia, drug delivery, or higher temperature ablations. We remain confident in the continued process of its successful clinical applications, from breast cancer screening to future therapies.

## Figures and Tables

**Table 1:** The quantitative measurements of common breast tissues for both sound speed and attenuation coefficients at 2.5 MHz in a phantom [37].

Material	Sound Speed [mm × µs^−1^]	Attenuation Coefficient at 2.5 MHz [dB/cm]
Fat	1.467	0.48
Parenchymal tissue	1.552	0.89
Cancer	1.563	1.20
Fibroadenoma	1.552	0.52
Gelatin cyst	1.585	0.16

**Table 2:** Comparison of ultrasound BI-RADS (* 5th edition) [7] classification parameters and proposed comparable SoftVue BTUS BI-RADS parameters (bolded). Abbreviations: BTUS, breast tomographic ultrasound; REF, Reflection; SS, sound speed.

ULTRASOUND BI-RADSs *				BTUS BI-RADSs * (Under Development)		
**Tissue Composition (screening only)**	a. Homogeneous—fat			**Volume Averaged Sound Speed**	**a. Fatty**	
	b. Homogeneous—fibroglandular			**Like Mammography**	**b. Scattered**	
	c. Heterogeneous echotexture				**c. Heterogenously Dense**	
					**d. Extremely Dense**	
**Masses**	**Shape**	**Oval**		**Oval**		
		**Round**		**Round**		
		**Irregular**		**Irregular**		
	Orientation	Parallel				
		Not parallel				
	**Margin**	**Circumscribed**		**Circumscribed**		
		**Not circumscribed**	**Indistinct**	**Not circumscribed**	**Indistinct**	
			**Angular**			
			**Microlobulated**			
			**Spiculated**		**Spiculated**	
	Echo pattern	Anechoic		**Black (Reflection)**		
		Hyperechoic		**Bright/White (Reflection)**		
		Complex cystic and solid				
		Hypoechoic		**Dark (Reflection)**		
		Isoechoic		**Gray (Reflection)**		
		Heterogeneous				
	Posterior features	No posterior features				
		**Enhancement**		Blue * (Stiffness Fusion contains low attenuation)		
		Shadowing *		Red * (Stiffness Fusion contains high attenuation)		
		Combined pattern				
Calcifications	In a mass			**(Resolution limited)**		
	Outside a mass					
	Intraductal calcifications					
**Associated features**	**Architectural distortion**			**Prominent in Coronal (REF and SS)**		
	**Duct changes**			**(Resolution limited)**		
	**Skin changes**	**Skin-thickening**		**(Angle dependent)**		
		**Skin retraction**		**Prominent in Coronal (REF and SS)**		
	**Edema**			**Seen in REF and SS**		
	Vascularity	Absent		**(Not currently available)**		
		Internal vascularity				
		Vessels in rim				
	**Elasticity Assessment**	**Soft**		**Black-Blue (Stiffness Fusion)**		
		**Intermediate**		**Green-Yellow (Stiffness Fusion)**		
		**Hard**		**Orange-Red (Stiffness Fusion)**		

**Table 3:** SoftVue findings with its associated BTUS BI-RADSs (* under development).

SoftVue Findings	BTUS * BI-RADSs	
Negative	1	Negative
Circumscribed + Blue	2	Benign
Circumscribed + Green	3	Probably Benign
Circumscribed + Orange/Red	4a	Low Suspicion for Malignancy
Indistinct + Green	4b	Moderate Suspicion
Indistinct/Irregular + Orange/Red	4c	High Suspicion
Spiculations/AD + Orange/Red	5	High Suspicion

## Data Availability

The data presented in this study are available on request from the corresponding author.

## Abbreviations

AI, artificial intelligence; BI-RADS, breast imaging reporting and data systems; BTUS, breast tomographic ultrasound; CEM, contrast-enhanced mammography; CPU, computer processing unit; DMT, Delphinus Medical Technologies, Incorporated; FDA, Food and Drug Administration; FGI, fat–glandular interface; FFDM, full field digital mammography; GPU, graphics processing unit; HHUS, handheld ultrasound; ICDR, incremental cancer detection rate; MR, magnetic resonance; MHz, megahertz; MBI, molecular breast imaging; PMA, Pre-Marketing Approval; REF, Reflection; SS, sound speed; TR, time-reversed; US, ultrasound.
